# Supplementary material for: Identifying new cancer genes based on the integration of annotated gene sets via hypergraph neural networks
Source: Bioinformatics. 2024 Jun 28;40(Suppl 1):i511–20. doi: 10.1093/bioinformatics/btae257 (PMC11211849; doi:10.1093/bioinformatics/btae257)
Supplement: btae257_Supplementary_Data [file btae257_supplementary_data.pdf]

Supplementary Material for

Identifying new cancer genes based on the integration of annotated gene sets via hypergraph neural networks

The following pages contain supplementary methods, supplementary results, nine supplementary figures, and fourteen supplementary tables that support the findings of the main paper.

Supplementary Methods

1.1 Compared methods

We conduct a comparison with five state-of-the-art cancer gene prediction methods, namely 20/20+, DORGE, EMOGI, MTGCN and NetCore. Furthermore, to emphasize the advantages and necessity of employing the hypergraph for representing annotated gene sets, we compare DISHyper with two graph-based representation methods, including GCN and GCNII. A brief introduction to these comparison methods is as follows: (1) 20/20+ designed a series of mutation-based feature-trained random forest models to find genes with the same mutation pattern as known cancer genes. (2) DORGE combined mutation data with epigenetic data and added epigenetic features such as methylation, histone modifications, and super-enhancer percentages on the basis of 20/20+. (3) EMOGI proposed integrating multi-omics features based on the graph convolution network to identify new cancer genes by learning local neighborhood features of cancer genes. (4) MTGCN added the linkage prediction auxiliary task to the PPI network on the basis of EMOGI and optimized the learning of gene features through this multi-task learning framework. (5) NetCore is a novel network propagation approach based on node coreness. (6) GCN: We utilize graph structure to represent annotated gene sets and apply GCN for gene feature extraction. Specifically, we consider genes as nodes, represent each annotated gene set as a complete subgraph, and combine all complete subgraphs into a graph. We use this graph to represent all annotated gene sets and as input for the GCN. (7) GCNII: This variant incorporates initial residual and identity mapping into the GCN, which offers enhanced model expressiveness and utilizes the same inputs as GCN.

1.2 CRISPR loss-of-function screening experiment results

We use CRISPR loss-of-function screening experiment results from the DepMap database (released on December 22, 2022). The DepMap database contains the results of genome-scale CRISPR knockout screens for project Achilles and SCORE . The Broad Institute has developed Chronos to assess the impact of individual gene expression on the survival and proliferation of tumor cell lines in CRISPR loss-of-function screening experiments . They have compiled a cancer dependency map between genes and tumor cell survival using over 1000 genetically characterized cancer cell lines. Specifically, the Broad Institute calculates a Chronos score for each gene in each tumor cell line based on the CRISPR loss-of-function screening results, which represents the impact of genes on cell proliferation in the functional loss-of-function screening. Based on the distribution of Chronos scores for each gene, we define genes with a Chronos score < - 0.5 as essential genes in the tumor cell lines. Furthermore, we define genes with a Chronos score < - 0.5 in most cell lines as globally essential genes. We use these globally essential genes for validating DISHyper prediction results. We calculate the enrichment of DISHyper prediction results in globally essential genes using Fisher's exact test and compare them with known cancer genes, neutral genes, and randomly selected genes. For the other three gene sets, we randomly select 200 genes from each set and calculate their enrichment in globally essential genes. We repeat this random process over 500 times and compute the average p-value.

1.3 Statistical analysis

Binomial test is used to test the enrichment of cancer genes annotated by cancerMine in the first decile of the DISHyper prediction results. Gene enrichment analyses are using one-sided Fisher’s exact test.

Supplementary Results

2.1 The model ablation analysis for DISHyper

To explore the effect of the disease-specific hyperedge weighting module and the hypergraph residual learning module, we compare two variants of DISHyper. (1) HGNN: This variant has fully connected layers with the same number of layers and dimensions as in DISHyper but replaces the three hypergraph residual learning modules with a two-layer hypergraph neural network. (2) ResHGNN: This variant has the same model structure as DISHyper but without the disease-specific hyperedge weighting module.

We investigate the effect of the hypergraph residual learning module by comparing HGNN with ResHGNN. Just as using residual structures in GCN, we aim to alleviate the over-smoothing phenomenon that occurs in multiple-layer HGNN through it (Supplementary Methods). As shown in Supplementary Table 4, ResHGNN outperforms HGNN, with improvements of 1.1% and 1.2% on AUROC and AUPRC, respectively. We also investigate the effect of the disease-specific hyperedge weighting module by comparing ResHGNN with DISHyper. We can use this module to screen for crucial information in different types of annotated gene sets and reduce the impact of noise. According to the results in Supplementary Table S6, the disease-specific hyperedge weighting module can significantly improve the performance of the model, and the module improves by 1.3% and 2.1% on AUROC and AUPRC, respectively. The above results illustrate the effectiveness of our proposed two modules, which help us to predict cancer genes more accurately.

Moreover, to examine the impact of the use of hypergraphs on predictions, we compared the performance of GCN and HGNN. In GCN, we represent annotated gene sets using a graph and apply GCN for gene feature extraction. Specifically, we consider genes as nodes, represent each annotated gene set as a complete subgraph, and combine all complete subgraphs into a graph. We found that HGNN outperforms GCN on both performance metrics (Table S14). The result may indicated that the use of hypergraphs also plays a role in improving the performance of DISHyper. DISHyper achieves the best results by combining HGNN with hyperedge weighting.

2.2 A case study to show the interpretability of the DISHyper prediction process

DISHyper integrates knowledge from different types of annotated gene sets to predict cancer genes, which may offer valuable insights into the underlying pathogenic mechanisms. As outlined in the preceding sections, WNT5A emerges as a predicted cancer gene uniquely identified by DISHyper, setting it apart from other methodologies. This case study delves into the DISHyper prediction process, focusing on WNT5A.

WNT5A is a protein-coding gene that belongs to the Wnt family and signals through both classical and non-classical WNT pathways. In our dataset, WNT5A is associated with 957 annotated gene sets, each assigned a weight to denote its relevance to cancer gene prediction. Examination of the top 100 hyperedge-weighted annotated gene sets reveals a multitude of biological processes linked to tumorigenesis. Notable processes include breast epithelial cell proliferation, mesenchymal cell proliferation, fibroblast proliferation, and other cell proliferation processes, epithelial to mesenchymal transition, mammary duct morphogenesis, and other biological processes closely related to cell invasion and metastasis. Additionally, processes such as immune and cell apoptosis processes such as lymphocyte apoptosis, leukocyte apoptosis, and thymocyte apoptosis are evident (Supplementary Figure S6). We use hypergraphs to represent the higher-order association of WNT5A with known cancer genes and other genes in these cancer-related biological processes. We extract higher-order functional association information for WNT5A within the annotated gene set through a two-stage message-passing process in the hypergraph neural network. This process culminates in the identification of WNT5A as a cancer gene (Supplementary Figure S6). WNT5A has been identified as a driver gene for a variety

of cancers such as breast cancer and melanoma. The above biological processes may be the crucial reason why WNT5A affects the occurrence and development of breast cancer and melanoma.

Supplementary Figures

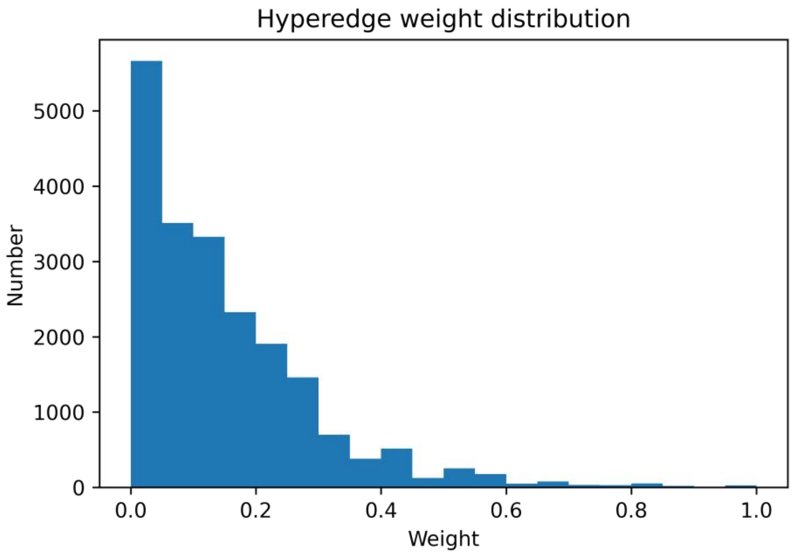

Figure S1. The distribution of hyperedge weights.

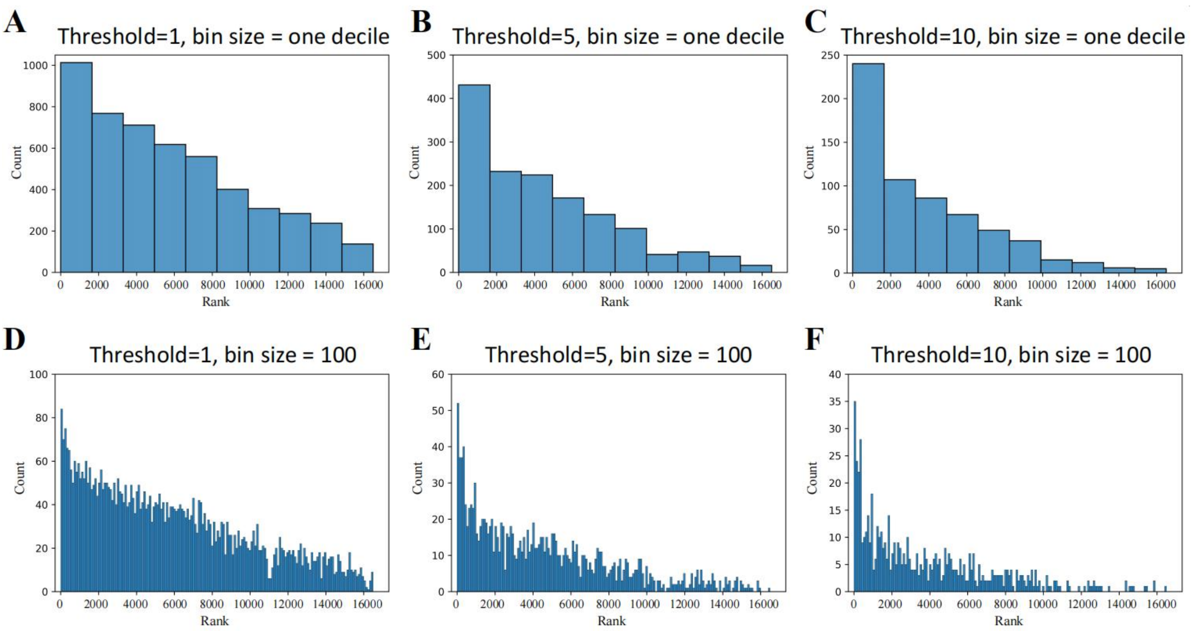

Figure S2. Analysis of DISHyper ranking results using cancer genes screened by different thresholds in the cancerMine database, where the Threshold =5 indicates that the gene was reported to be associated with cancer in more than 5 papers. (A-C) Distribution of screened cancer genes in each decile of DISHyper ranking results. The cancer genes are significantly enriched in the first decile, and the number of cancer genes enriched in the first decile is significantly higher than in the other deciles. (D-F) The number of cancer genes per 100 genes in DISHyer ranking results. The top-ranked 100 genes contained the highest number of cancer genes of all bins, and the number of cancer genes decreased significantly as the ranking increased.

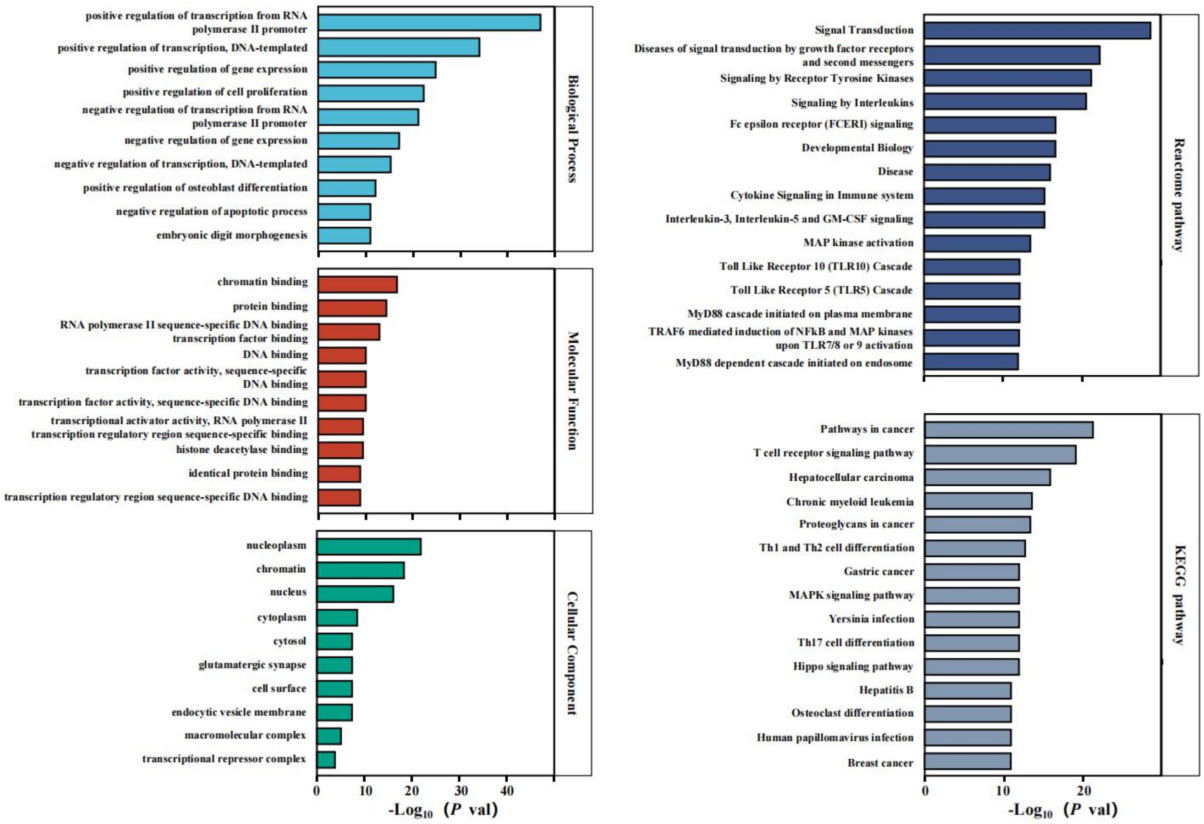

Figure S3. Bar graph representing the biological process, molecular function, cellular component, Reactome pathway, and KEGG pathway functional enrichment analysis of DISHyper-predicted cancer genes using DAVID.

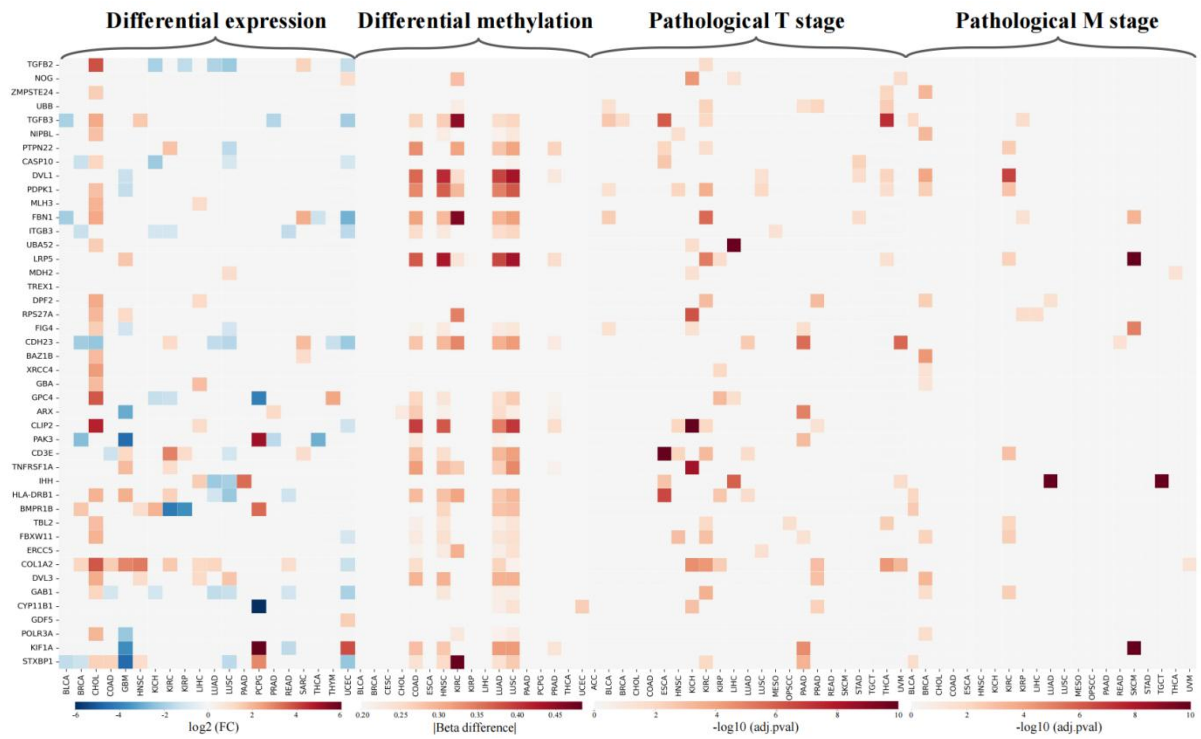

Figure S4. Validation of 44 novel cancer genes (novelCG) utilizing gene expression and DNA methylation data across various tumor samples from the TCGA study. The validation process includes assessing the association between novelCG and different cancers through differential gene expression analysis, differential methylation, and examining differential gene expression at the pathological diagnosis stage. Significantly altered genes are represented by dots in the figure.

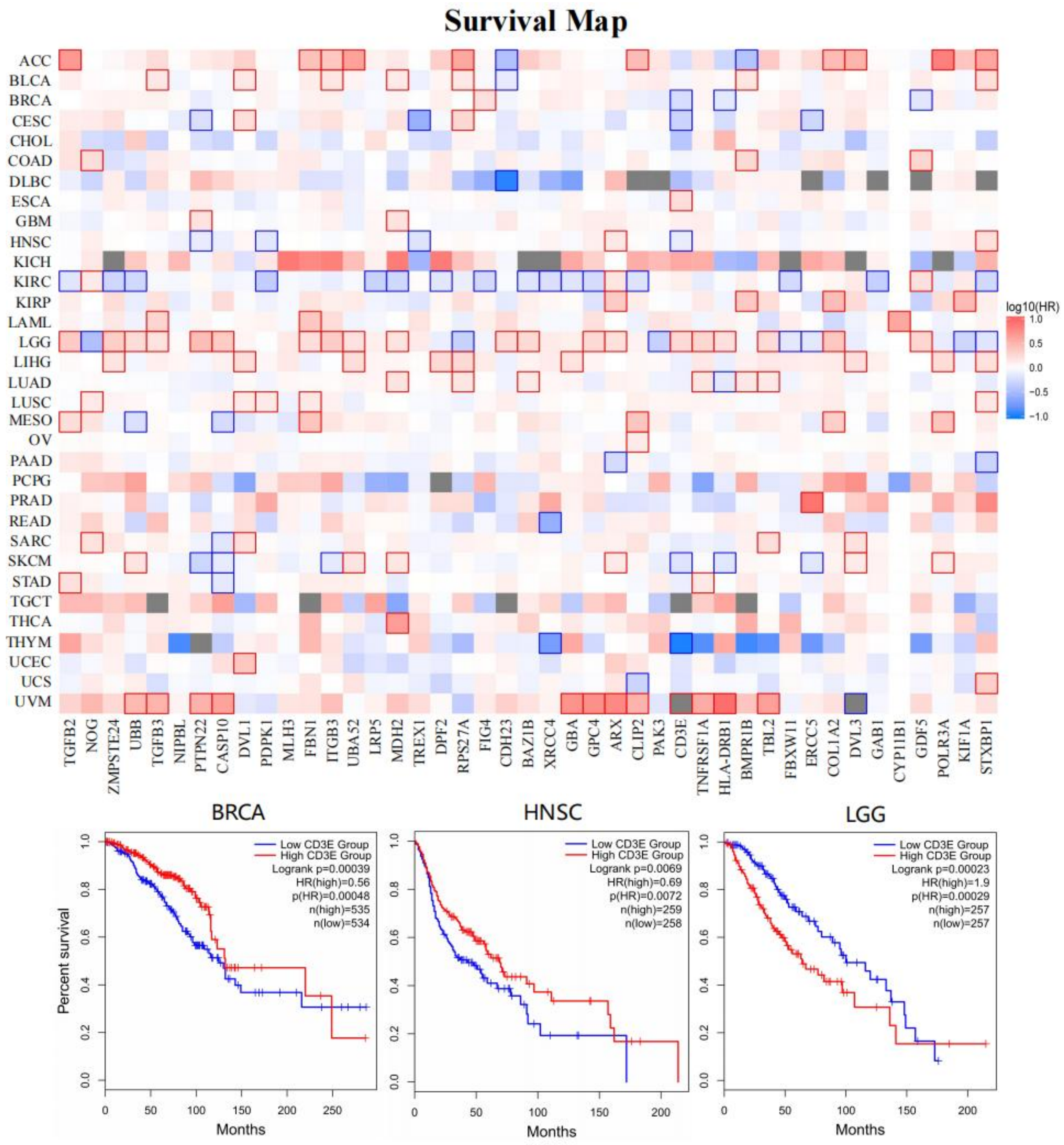

Figure S5. Results of survival analysis for 44 novel cancer genes (novelCG) across 33 cancer types. Points outlined in red or blue highlight genes whose expression significantly impacts the survival duration of specific cancers. At the bottom of the figure, detailed survival analysis curves for CD3E are presented in three cancer types: BRCA, HNSC, and LGG.

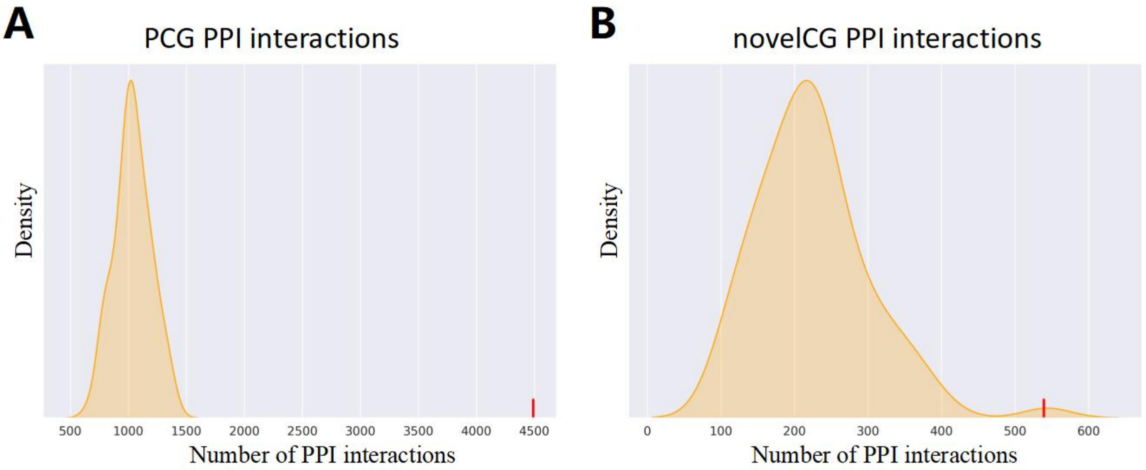

Figure S6. Analyze the network properties of DISHyper-predicted PCG and novelCG based on the STRING PPI network. Both PCG and novelCG would have significant numbers of interactions with known cancer genes on the PPI network. In each plot, the red vertical line indicates the number of interactions between predictions and KCG. The yellow curve shows the distribution of the number of interactions between randomly selected genes and KCG. We repeat 10,000 experiments, and each experiment randomly selects the same number of genes as PCG or novelCG to statistic their interactions with KCG.

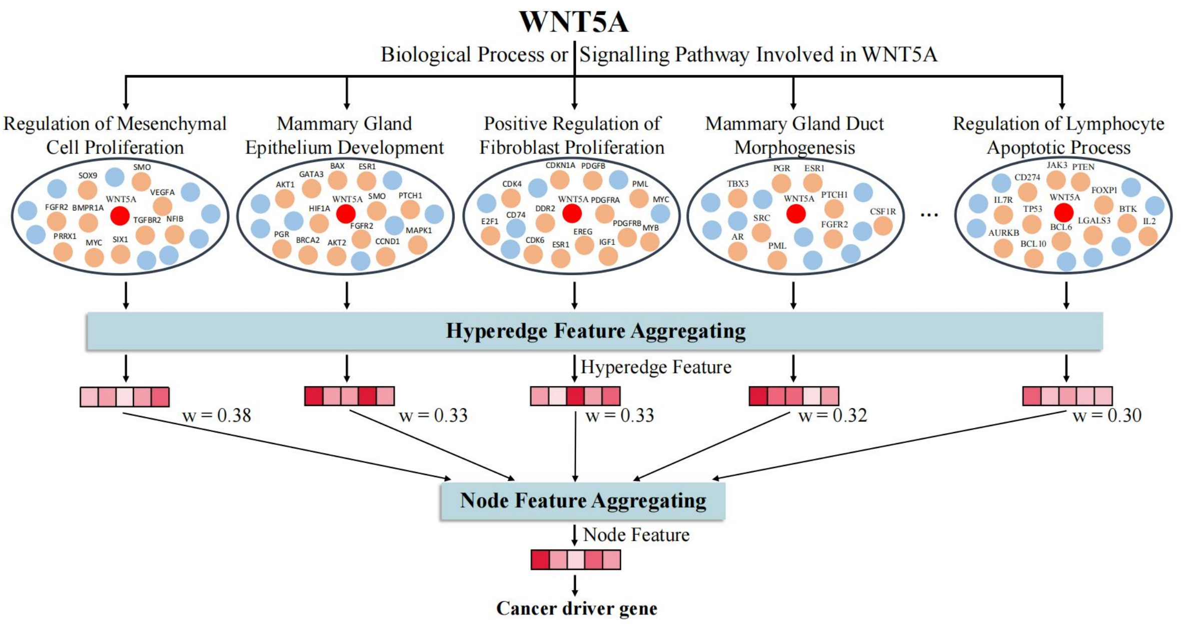

Figure S7. Illustration of the DISHyper prediction process. We illustrate of the DISHyper prediction process with a case study of *WNT5A*. *WNT5A* participates in more than 900 annotated gene sets. DISHyper generates annotated gene set (hyperedge) features through the hyperedge feature aggregation process and then weighted aggregation of hyperedge features through the node feature aggregation process and generates feature representations of *WNT5A*. Finally, DISHyper predicts *WNT5A* as a cancer gene based on the known cancer gene feature patterns and the features of *WNT5A*. There will be multiple node-hyperedge-node feature aggregation processes in DISHyper through which more node neighbor information is extracted. The w in the figure represents the weight of the annotation gene set (hyperedge), where a larger weight indicates that more information from the annotation gene set will be aggregated into the gene features. The figure only shows the five biological processes with representative and large weights.

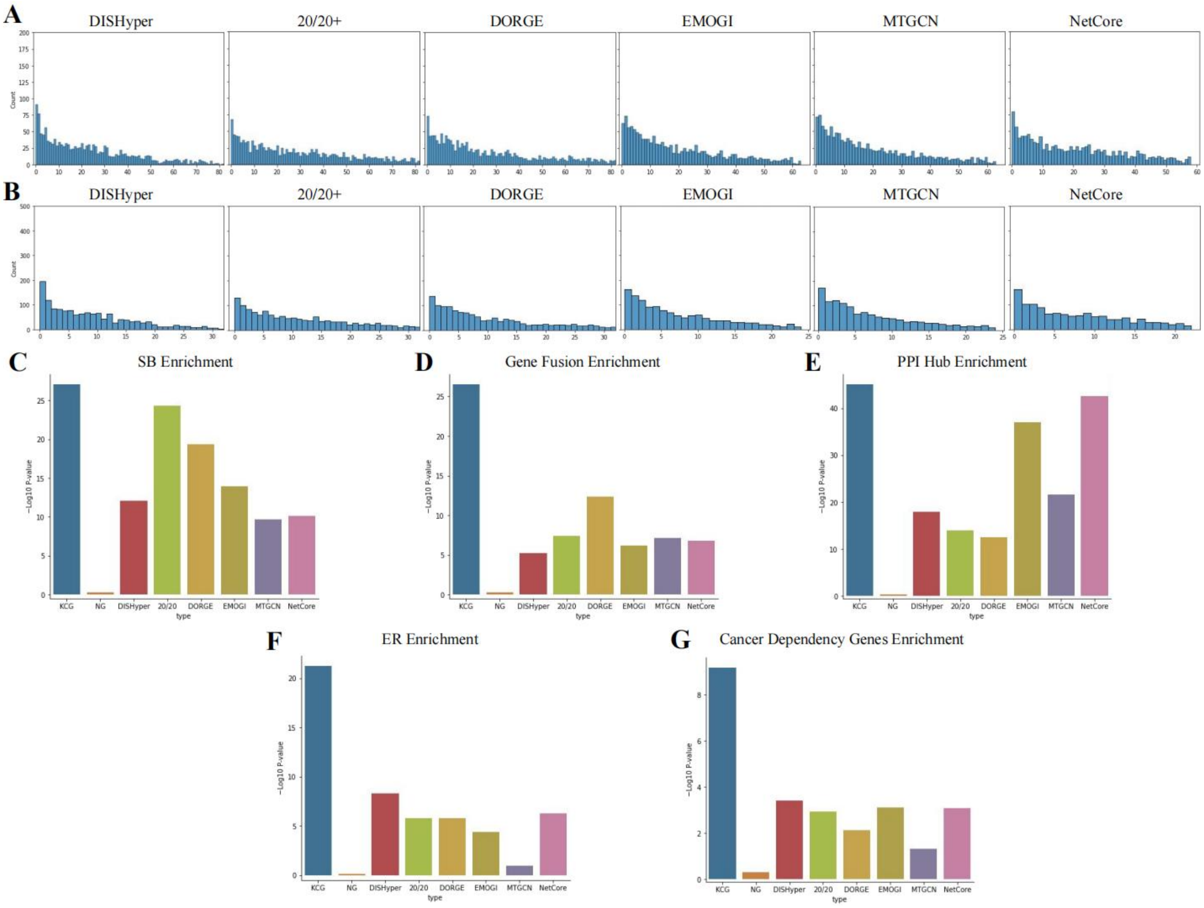

Figure S8. Enrichment analysis results of other cancer gene prediction methods on multiple data. (A) Validation of the ranking results of DISHyper based on the cancerMine database. We show the number of genes annotated as cancer genes by the CancerMine database per 200 genes in the ranking results of each method. (B) The number of genes annotated as cancer genes by the CancerMine database per 500 genes in the ranking results of each method. We find that DISHyper enriched more cancerMine annotated cancer genes in both top-ranked 200 and top-ranked 500. Enrichment analysis of the top-rank 200 genes of each method in SB inactivating pattern gene list (C), gene fusion list (D), BioGRID PPI network hub gene list (E), ER gene list (F), and essential cancer dependency genes (G). Different methods will exhibit different characteristics on functional genomic data due to their method principles. In order to compare the enrichment results of different methods, we adjusted the background genes, so this result may be partially inconsistent with the manuscript.

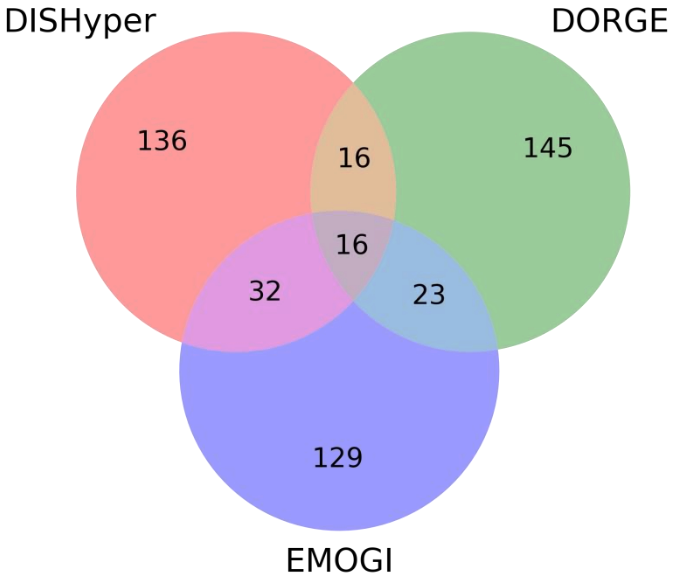

Figure S9. The relationship between the top-ranked 200 prediction results of the three methods DISHyper, DORGE and EMOGI.

Supplementary Tables

Table S1 The DISHyper-predicted top-ranked 200 cancer genes (after excluding the known cancer genes).

Rank	gene symbol	Rank	gene symbol	Rank	gene symbol
1	MAPK3	40	ERCC6	79	BMP7
2	GRB2	41	PIK3R2	80	PTK2
3	RELA	42	CRKL	81	ROR2
4	NFKB1	43	MAP3K7	82	CASP10
5	SOS1	44	IKBKG	83	JAG1
6	BMP4	45	TBX1	84	SMC3
7	FOS	46	ADA	85	MECP2
8	WNT5A	47	ELK1	86	ITGB1
9	CDC42	48	GREM1	87	DVL1
10	IFNG	49	TET2	88	PPP3CA
11	SHC1	50	SFRP1	89	PDPK1
12	GLI3	51	GJA1	90	MLH3
13	PRKCD	52	NKX2-5	91	FBN1
14	FYN	53	MEF2C	92	MED1
15	NFKBIA	54	APC2	93	SMARCC2
16	PIK3CD	55	PAK1	94	UBC
17	PRKCA	56	KDM1A	95	RFC2
18	CDKN2B	57	PIK3CG	96	PSMD12
19	LYN	58	RBPJ	97	ITGB3
20	TWIST1	59	TCF4	98	UBA52
21	BMP2	60	WNT4	99	LRP5
22	STAT1	61	ACTB	100	SFRP2
23	DKK1	62	CHD7	101	MDH2
24	SHH	63	HDAC1	102	CTBP1
25	TGFB2	64	SOX11	103	TREX1
26	NOG	65	KIF1B	104	DPF2
27	FGF10	66	CDH2	105	RPS27A
28	GSK3B	67	ZMPSTE24	106	SOX4
29	TGFBR1	68	UBB	107	SETD5
30	GATA4	69	MSH3	108	FIG4
31	SRF	70	TGFB3	109	TFAP2A
32	LIG4	71	ENG	110	CDH23
33	MAPK14	72	NIPBL	111	IRS1
34	STAT5A	73	LIMK1	112	RUNX2
35	INSR	74	CEBPB	113	BAZ1B
36	HES1	75	PTPN22	114	GTF2I
37	IGF2	76	NR3C1	115	XRCC4
38	CHUK	77	HDAC4	116	SEC23B
39	AIP	78	WNT3A	117	GBA

118	ACTG1	146	PAK3	174	KLLN
119	GPC4	147	DACT1	175	ACTL6B
120	YAP1	148	TMEM127	176	CYP11B1
121	HDAC8	149	CD3E	177	FOXP3
122	PTK2B	150	TNFRSF1A	178	GDF5
123	ARX	151	IHH	179	IL2RG
124	DLST	152	SNAI2	180	NRIP1
125	BPTF	153	GTF2IRD1	181	NFATC1
126	CLIP2	154	RAD51C	182	RBBP8
127	ZMIZ1	155	HLA-DRB1	183	POLR3A
128	DLK1	156	GATA6	184	BUB1
129	SOX10	157	BMPR1B	185	SP1
130	SATB2	158	YES1	186	RARB
131	PSEN1	159	SALL1	187	KIF1A
132	GDNF	160	PPP2CA	188	NOD2
133	CTLA4	161	PUF60	189	VCP
134	NOTCH3	162	RPS6KA3	190	MAPT
135	ROBO1	163	DYRK1A	191	SIN3A
136	ID2	164	TBL2	192	IRS2
137	YY1	165	SLC25A11	193	GLI2
138	SIRT1	166	ZAP70	194	ACTL6A
139	SOS2	167	FBXW11	195	SRY
140	RERE	168	GAB2	196	HNF4A
141	IL2RA	169	IL6R	197	SMARCA2
142	RASA1	170	ERCC5	198	HBEGF
143	SOD1	171	COL1A2	199	STXBP1
144	RASGRP1	172	DVL3	200	TBC1D24
145	WNT1	173	GAB1		

Table S2 The DISHyper-predicted 44 novel cancer genes.

Rank	gene symbol	Rank	gene symbol
25	TGFB2	115	XRCC4
26	NOG	117	GBA
67	ZMPSTE24	119	GPC4
68	UBB	123	ARX
70	TGFB3	126	CLIP2
72	NIPBL	146	PAK3
75	PTPN22	149	CD3E
82	CASP10	150	TNFRSF1A
87	DVL1	151	IHH
89	PDPK1	155	HLA-DRB1
90	MLH3	157	BMPR1B
91	FBN1	164	TBL2
97	ITGB3	167	FBXW11
98	UBA52	170	ERCC5
99	LRP5	171	COL1A2
101	MDH2	172	DVL3
103	TREX1	173	GAB1
104	DPF2	176	CYP11B1
105	RPS27A	178	GDF5
108	FIG4	183	POLR3A
110	CDH23	187	KIF1A
113	BAZ1B	199	STXBP1

Table S3 The annotated gene sets used in DISHyper

MSigDB collection	Category	Number
C2	Chemical and genetic perturbations	2884
	Pathways	2837
C5	GO	10185
	HPO	4721

Table S4 P-values of Wilcoxon rank sum test for DISHyper versus other methods on two performance evaluation metrics.

Method	P-values of AUROC	P-values of AUPRC
20/20+	$< 6.6\times10^{-10}$	$< 6.6\times10^{-10}$
DORGE	2.4×10^{-9}	1.3×10^{-8}
EMOGI	7.5×10^{-10}	9.5×10^{-10}
MTGCN	9.5×10^{-10}	1.5×10^{-9}
NetCore	$< 6.6\times10^{-10}$	$< 6.6\times10^{-10}$
GCN	2.3×10^{-7}	2.6×10^{-8}
GCNII	2.4×10^{-5}	8.6×10^{-7}

Table S5 Comparative experiment between node degree of weighted hypergraph* and DISHyper.

Method	AUROC	AUPRC
DISHyper (node degree)	0.918 ± 0.014	0.857 ± 0.022
DISHyper	0.937 ± 0.014	0.894 ± 0.019

*Node degree of weighted hypergraph: We assign a score to each gene as the degree of the weighted hypergraph and evaluate the performance of it using five-fold cross-validation.

Table S6 Results of model ablation experiments

Model	AUROC	AUPRC
HGNN	0.914 ± 0.016	0.861 ± 0.021
ResHGNN	0.924 ± 0.015	0.873 ± 0.022
DISHyper (Ours)	0.937 ± 0.014	0.894 ± 0.019

Table S7 The enrichment results of DISHyper's prediction results in KEGG and REACTOME pathway gene sets were analyzed based on GSEA.

Name	Size	FDR
REACTOME_REGULATION_OF_SIGNALING_BY_CBL	16	0
REACTOME_SIGNALING_BY_ERYTHROPOIETIN	15	5.00E-04
REACTOME_DOWNSTREAM_SIGNALING_EVENTS_OF_B_CELL_RECEPTOR_BCR	69	3.33E-04
REACTOME_DEGRADATION_OF_GLI1_BY_THE_PROTEASOME	56	2.50E-04
KEGG_MEDICUS_VARIANT_MUTATION_INACTIVATED_VCP_TO_26S_PROTEASOME_MEDIATED_PROTEIN_DEGRADATION	39	2.00E-04
REACTOME_STABILIZATION_OF_P53	49	3.33E-04
KEGG_MEDICUS_VARIANT_MUTATION_CAUSED_ABERRANT_SOD1_TO_26S_PROTEASOME_MEDIATED_PROTEIN_DEGRADATION	40	2.85E-04

KEGG_MEDICUS_REFERENCE_26S_PROTEASOME_MEDIATED_PROTEIN_DEGRADATION	38	2.50E-04
KEGG_MEDICUS_VARIANT_MUTATION_INACTIVATED_UBQLN2_TO_26S_PROTEASOME_MEDIATED_PROTEIN_DEGRADATION	39	3.33E-04
REACTOME_DEFECTIVE_CFTR_CAUSES_CYSTIC_FIBROSIS	60	3.00E-04
KEGG_MEDICUS_VARIANT_MUTATION_CAUSED_ABERRANT_ABETA_TO_26S_PROTEASOME_MEDIATED_PROTEIN_DEGRADATION	35	2.73E-04
REACTOME_SOMITOGENESIS	51	2.50E-04
REACTOME_RUNX1_REGULATES_TRANSCRIPTION_OF_GENES_INVOLVED_IN_DIFFERENTIATION_OF_HSCS	57	2.31E-04
REACTOME_DEGRADATION_OF_DVL	55	2.14E-04
REACTOME_NEGATIVE_REGULATION_OF_NOTCH4_SIGNALING	51	2.00E-04
KEGG_MEDICUS_VARIANT_MUTATION_CAUSED_ABERRANT_SNCA_TO_26S_PROTEASOME_MEDIATED_PROTEIN_DEGRADATION	35	1.87E-04
KEGG_MEDICUS_VARIANT_SCRAPIE_CONFORMATION_PRPSC_TO_26S_PROTEASOME_MEDIATED_PROTEIN_DEGRADATION	35	1.76E-04
REACTOME_SCF_BETA_TRCP_MEDIATED_DEGRADATION_OF_EMI1	54	1.67E-04
REACTOME_DEGRADATION_OF_AXIN	52	1.58E-04
KEGG_MEDICUS_VARIANT_MUTATION_CAUSED_ABERRANT_HTT_TO_26S_PROTEASOME_MEDIATED_PROTEIN_DEGRADATION	35	1.50E-04
REACTOME_AUF1_HNRNP_D0_BINDS_AND_DESTABILIZES_MRNA	53	1.43E-04
REACTOME_NEGATIVE_REGULATION_OF_FGFR3_SIGNALING	19	1.36E-04
REACTOME_REGULATION_OF_RAS_BY_GAPS	62	1.30E-04
REACTOME_REGULATION_OF_RUNX3_EXPRESSION_AND_ACTIVITY	48	1.25E-04
KEGG_PROTEASOME	43	1.20E-04
KEGG_COLORECTAL_CANCER	23	1.54E-04
REACTOME_CROSS_PRESENTATION_OF_SOLUBLE_EXOGENOUS_ANTIGENS_ENDOSOMES	48	1.48E-04
REACTOME_HEDGEHOG_LIGAND_BIOGENESIS	63	1.43E-04
KEGG_GLYCOSAMINOGLYCAN_BIOSYNTHESIS_CHONDROITIN_SULFATE	21	1.38E-04
REACTOME_DEGRADATION_OF_BETA_CATENIN_BY_THE_DESTRUCTION_COMPLEX	74	1.33E-04
REACTOME_DECTIN_1_MEDIATED_NONCANONICAL_NF_KB_SIGNALING	59	1.61E-04
REACTOME_ASYMMETRIC_LOCALIZATION_OF_PCP_PROTEINS	63	2.19E-04
KEGG_ERBB_SIGNALING_PATHWAY	47	2.42E-04
REACTOME_METABOLISM_OF_POLYAMINES	56	2.35E-04
REACTOME_SIGNALING_BY_FGFR3	24	2.28E-04
REACTOME_REGULATION_OF_RUNX2_EXPRESSION_AND_ACTIVITY	69	2.22E-04
REACTOME_SIGNALING_BY_NTRK2_TRKB	16	2.70E-04
REACTOME_NEGATIVE_REGULATION_OF_FGFR2_SIGNALING	24	2.89E-04
REACTOME_SIGNALING_BY_CSF1_M_CSF_IN_MYELOID_CELLS	22	2.82E-04
REACTOME_SCF_SKP2_MEDIATED_DEGRADATION_OF_P27_P21	52	3.25E-04
KEGG_ACUTE_MYELOID_LEUKEMIA	25	3.17E-04
REACTOME_FORMATION_OF_PARAXIAL_MESODERM	60	3.09E-04

REACTOME_REGULATION_OF_PTEN_STABILITY_AND_ACTIVITY	62	3.02E-04
REACTOME_TRANSCRIPTIONAL_REGULATION_BY_RUNX2	94	3.18E-04
REACTOME_DOWNSTREAM_SIGNALING_OF_ACTIVATED_FGFR2	19	3.11E-04
REACTOME_HEDGEHOG_ON_STATE	77	3.04E-04
REACTOME_NEGATIVE_REGULATION_OF_FGFR1_SIGNALING	22	2.98E-04
REACTOME_REGULATION_OF_MECP2_EXPRESSION_AND_ACTIVITY	21	3.33E-04
KEGG_CHRONIC_MYELOID_LEUKEMIA	33	3.47E-04
REACTOME_G1_S_DNA_DAMAGE_CHECKPOINTS	57	3.80E-04
KEGG_PRIMARY_IMMUNODEFICIENCY	28	3.92E-04
REACTOME_GASTRULATION	92	3.84E-04
KEGG_NON_SMALL_CELL_LUNG_CANCER	23	3.96E-04
REACTOME_CONSTITUTIVE_SIGNALING_BY_ABERRANT_PI3K_IN_CANCER	43	5.92E-04
REACTOME_ROS_AND_RNS_PRODUCTION_IN_PHAGOCYTES	33	6.91E-04
REACTOME_SIGNALING_BY_NOTCH4	71	7.14E-04
REACTOME_PI_3K_CASCADE_FGFR2	16	7.01E-04
REACTOME_RUNX2_REGULATES_BONE_DEVELOPMENT	20	7.24E-04
KEGG_ENDOMETRIAL_CANCER	19	7.79E-04
REACTOME_MAPK6_MAPK4_SIGNALING	73	8.00E-04
REACTOME_INTERLEUKIN_3_INTERLEUKIN_5_AND_GM_CSF_SIGNALING	32	8.19E-04
KEGG_RENAL_CELL_CARCINOMA	33	8.06E-04
REACTOME_PCP_CE_PATHWAY	87	8.09E-04
REACTOME_MET_PROMOTES_CELL_MOTILITY	34	8.28E-04
REACTOME_NOTCH1_INTRACELLULAR_DOMAIN_REGULATES_TRANSCRIPTION	35	9.23E-04
REACTOME_FRS_MEDIATED_FGFR2_SIGNALING	17	9.54E-04
REACTOME_SIGNALING_BY_FGFR1	35	9.70E-04
KEGG_SMALL_CELL_LUNG_CANCER	56	1.00E-03
REACTOME_SIGNALING_BY_THE_B_CELL_RECEPTOR_BCR	95	0.001014
REACTOME_SIGNALING_BY_CSF3_G_CSF	19	0.001042
KEGG_T_CELL_RECEPTOR_SIGNALING_PATHWAY	69	0.001126
REACTOME_MET_ACTIVATES_PTK2_SIGNALING	24	0.001138
REACTOME_TP53_REGULATES_TRANSCRIPTION_OF_DNA_REPAIR_GENES	37	0.001259
REACTOME_TCR_SIGNALING	104	0.001391
REACTOME_MAPK_TARGETS_NUCLEAR_EVENTS_MEDIATED_BY_MAP_KINASES	25	0.001479
KEGG_PANCREATIC_CANCER	29	0.001880
REACTOME_FC_EPSILON_RECEPTOR_FCERI_SIGNALING	112	0.001869
REACTOME_EPHB_MEDIATED_FORWARD_SIGNALING	38	0.001871
REACTOME_TRANSCRIPTIONAL_REGULATION_BY_RUNX3	70	0.002024
REACTOME_SYNDECAN_INTERACTIONS	19	0.001999
REACTOME_FCERI_MEDIATED_NF_KB_ACTIVATION	81	0.002098
REACTOME_SIGNALING_BY_FGFR4	25	0.002084

REACTOME_ABC_TRANSPORTER_DISORDERS	77	0.002131
REACTOME_ORC1_REMOVAL_FROM_CHROMATIN	69	0.002403
REACTOME_SIGNALING_BY_SCF_KIT	22	0.002563
REACTOME_FRS_MEDIATED_FGFR1_SIGNALING	15	0.002568
KEGG_PATHWAYS_IN_CANCER	179	0.002562
REACTOME_HEDGEHOG_OFF_STATE	104	0.002544
REACTOME_NEGATIVE_REGULATION_OF_FGFR4_SIGNALING	21	0.002538
KEGG_B_CELL_RECEPTOR_SIGNALING_PATHWAY	46	0.002576
REACTOME_GROWTH_HORMONE_RECEPTOR_SIGNALING	17	0.002559
REACTOME_ERK_MAPK_TARGETS	20	0.002553
KEGG_FC_EPSILON_RI_SIGNALING_PATHWAY	55	0.002547
REACTOME_HOMOLOGOUS_DNA_PAIRING_AND_STRAND_EXCHANGE	26	0.002605
REACTOME_CLEC7A_DECTIN_1_SIGNALING	87	0.002599
REACTOME_INACTIVATION_OF_CSF3_G_CSF_SIGNALING	16	0.002592
REACTOME_ATTACHMENT_AND_ENTRY	15	0.002607
REACTOME_ZBP1_DAI_MEDIATED_INDUCTION_OF_TYPE_I_IFNS	18	0.002682
REACTOME_CARDIOGENESIS	21	0.002655
REACTOME_APC_C_CDH1_MEDIATED_DEGRADATION_OF_CDC20_AND_OTHER_APC_C_CDH1_TARGETED_PROTEINS_IN_LATE_MITOSIS_EARLY_G1	69	0.002679

Table S8 The cancer-related gene fusion results of novelCG.

Gene symbol	Source	Cancer Type	Sample	Target gene
TGFB2	Gao et al	BRCA	TCGA-A2-A0D0-01A	VAMP7
ZMPSTE24	Gao et al	BLCA	TCGA-FD-A62S-01A	SMAP2
ZMPSTE24	Gao et al	BRCA	TCGA-A2-A4RW-01A	YTHDF2
ZMPSTE24	Gao et al	BRCA	TCGA-EW-A1P7-01A	RLF
ZMPSTE24	TumorFusions	COAD	TCGA-A6-5660-01A	TMCO2
ZMPSTE24	Gao et al	COAD	TCGA-A6-5660-01A	TMCO2
ZMPSTE24	TumorFusions	LIHC	TCGA-DD-A4NE-01A	ZBTB7B
ZMPSTE24	Gao et al	LIHC	TCGA-DD-A4NE-01A	ZBTB7B
ZMPSTE24	Gao et al	OV	TCGA-25-1628-01A	RNF220
ZMPSTE24	Gao et al	OV	TCGA-61-1736-01B	CAP1
ZMPSTE24	TumorFusions	PRAD	TCGA-CH-5788-01A	GPBP1L1
ZMPSTE24	Gao et al	PRAD	TCGA-CH-5788-01A	GPBP1L1
ZMPSTE24	TumorFusions	UCEC	TCGA-AJ-A3BD-01A	TMCO2
UBB	Gao et al	LUAD	TCGA-62-8395-01A	PGC
NIPBL	TumorFusions	BLCA	TCGA-ZF-AA4V-01A	MRPS30
NIPBL	TumorFusions	BLCA	TCGA-FJ-A3Z7-01A	SPEF2
NIPBL	Gao et al	BLCA	TCGA-FJ-A3Z7-01A	SPEF2
NIPBL	Gao et al	BLCA	TCGA-ZF-AA4V-01A	MRPS30
NIPBL	TumorFusions	BRCA	TCGA-A7-A13D-01B	CLSPN
NIPBL	TumorFusions	BRCA	TCGA-A7-A6VV-01A	SLC30A9
NIPBL	Gao et al	BRCA	TCGA-A2-A0T1-01A	ANKRD17

NIPBL	Gao et al	BRCA	TCGA-A7-A13D-01A	CLSPN
NIPBL	Gao et al	BRCA	TCGA-A7-A6VV-01A	SLC30A9
NIPBL	TumorFusions	CHOL	TCGA-ZH-A8Y6-01A	NACC1
NIPBL	Gao et al	CHOL	TCGA-ZH-A8Y6-01A	NACC1
NIPBL	TumorFusions	ESCA	TCGA-L5-A8NI-01A	CPLANE1
NIPBL	TumorFusions	HNSC	TCGA-CV-7418-01A	SPEF2
NIPBL	Gao et al	HNSC	TCGA-CV-7418-01A	SPEF2
NIPBL	Gao et al	KIRP	TCGA-B9-A5W9-01A	ANKRD33B
NIPBL	TumorFusions	LGG	TCGA-DU-6406-01A	DSCR3
NIPBL	Gao et al	LGG	TCGA-DU-6406-01A	DSCR3
NIPBL	TumorFusions	LUAD	TCGA-95-7043-01A	ATP2A2
NIPBL	TumorFusions	LUAD	TCGA-50-5946-01A	SLC1A3
NIPBL	TumorFusions	LUAD	TCGA-05-4426-01A	MYO10
NIPBL	Gao et al	LUAD	TCGA-05-4426-01A	MYO10
NIPBL	Gao et al	LUAD	TCGA-95-7043-01A	ATP2A2
NIPBL	TumorFusions	LUSC	TCGA-18-4083-01A	ARID1A
NIPBL	TumorFusions	LUSC	TCGA-77-8153-01A	MAPRE1
NIPBL	TumorFusions	LUSC	TCGA-22-4595-01A	FGF10
NIPBL	TumorFusions	LUSC	TCGA-63-A5MW-01A	STK24
NIPBL	Gao et al	LUSC	TCGA-18-4083-01A	ARID1A
NIPBL	Gao et al	LUSC	TCGA-22-4595-01A	FGF10
NIPBL	Gao et al	LUSC	TCGA-63-A5MW-01A	STK24
NIPBL	Gao et al	LUSC	TCGA-77-8153-01A	MAPRE1
NIPBL	Gao et al	LUSC	TCGA-85-8479-01A	KHDC1
NIPBL	TumorFusions	READ	TCGA-AG-3731-01A	ARNTL2
NIPBL	Gao et al	READ	TCGA-AG-3731-01A	ARNTL2
NIPBL	TumorFusions	SARC	TCGA-DX-AB2W-01A	L3MBTL4
NIPBL	TumorFusions	SARC	TCGA-DX-A8BM-01A	SPEF2
NIPBL	Gao et al	SARC	TCGA-DX-A8BM-01A	SPEF2
NIPBL	Gao et al	SARC	TCGA-DX-AB2W-01A	L3MBTL4
NIPBL	TumorFusions	SKCM	TCGA-EE-A2MJ-06A	SLC1A3
NIPBL	TumorFusions	SKCM	TCGA-GN-A264-06A	SPEF2
NIPBL	TumorFusions	SKCM	TCGA-EE-A20I-06A	BASP1
NIPBL	TumorFusions	SKCM	TCGA-D3-A1Q3-06A	NUP155
NIPBL	Gao et al	SKCM	TCGA-D3-A1Q3-06A	ROPN1L
NIPBL	Gao et al	SKCM	TCGA-EE-A20I-06A	BASP1
NIPBL	Gao et al	SKCM	TCGA-GN-A264-06A	SPEF2
NIPBL	TumorFusions	UCEC	TCGA-AX-A3G4-01A	SPEF2
NIPBL	Gao et al	UCEC	TCGA-AX-A3G4-01A	SPEF2
NIPBL	TumorFusions	UCS	TCGA-NF-A4WX-01A	SPEF2
NIPBL	Gao et al	UCS	TCGA-NF-A4WX-01A	SPEF2
CASP10	TumorFusions	LUAD	TCGA-71-8520-01A	CFLAR
CASP10	TumorFusions	SKCM	TCGA-FS-A1ZJ-06A	EXOC6B
DVL1	Gao et al	CESC	TCGA-C5-A902-01A	SDF4

DVL1	Gao et al	ESCA	TCGA-L5-A8NE-01A	CCNL2
DVL1	Gao et al	GBM	TCGA-76-4926-01B	ACAP3
DVL1	Gao et al	LUAD	TCGA-93-A4JO-01A	CD2AP
DVL1	TumorFusions	LUSC	TCGA-96-A4JK-01A	ACAP3
DVL1	Gao et al	LUSC	TCGA-96-A4JK-01A	ACAP3
DVL1	Gao et al	OV	TCGA-31-1953-01A	ITPA
DVL1	TumorFusions	SARC	TCGA-DX-A7EU-01A	PLCH2
DVL1	TumorFusions	UCS	TCGA-NA-A4R0-01A	OPTN
DVL1	Gao et al	UCS	TCGA-NA-A4R0-01A	OPTN
PDPK1	TumorFusions	BRCA	TCGA-AR-A0TP-01A	CCNF
PDPK1	Gao et al	BRCA	TCGA-AR-A0TP-01A	CCNF
PDPK1	Gao et al	CESC	TCGA-FU-A3TQ-01A	UBALD1
PDPK1	Gao et al	KIRC	TCGA-B0-5692-01A	FBXO7
PDPK1	Gao et al	OV	TCGA-04-1347-01A	PRSS21
PDPK1	Gao et al	SKCM	TCGA-D3-A2JD-06A	HN1L
FBN1	TumorFusions	LGG	TCGA-DB-5277-01A	THSD4
FBN1	Gao et al	LGG	TCGA-DB-5277-01A	THSD4
FBN1	TumorFusions	MESO	TCGA-TS-A8AF-01A	TEX9
FBN1	Gao et al	MESO	TCGA-TS-A8AF-01A	TEX9
FBN1	TumorFusions	PRAD	TCGA-EJ-7783-01A	LEO1
FBN1	Gao et al	PRAD	TCGA-EJ-7783-01A	LEO1
FBN1	TumorFusions	SARC	TCGA-FX-A3TO-01A	PYGO1
FBN1	TumorFusions	SARC	TCGA-SG-A849-01A	EIF3B
FBN1	TumorFusions	SARC	TCGA-LI-A9QH-01A	C19orf2
FBN1	Gao et al	SARC	TCGA-DX-A8BT-01A	NSD2
FBN1	Gao et al	SARC	TCGA-FX-A3TO-01A	MYO5A
FBN1	Gao et al	SARC	TCGA-FX-A3TO-01A	PYGO1
FBN1	Gao et al	SARC	TCGA-IE-A6BZ-01A	COX14
FBN1	Gao et al	SARC	TCGA-LI-A9QH-01A	URI1
FBN1	Gao et al	SARC	TCGA-SG-A849-01A	EIF3B
ITGB3	Gao et al	BRCA	TCGA-A2-A0CX-01A	GRB7
ITGB3	Gao et al	OV	TCGA-04-1514-01A	UTP18
ITGB3	Gao et al	OV	TCGA-25-2401-01A	MYL4
UBA52	Gao et al	BRCA	TCGA-AO-A129-01A	KXD1
UBA52	Gao et al	BRCA	TCGA-E2-A14Z-01A	KDM4B
UBA52	Gao et al	LUAD	TCGA-55-1596-01A	CACNA1C
UBA52	Gao et al	STAD	TCGA-VQ-A8PE-01A	ARMC6
LRP5	Gao et al	ACC	TCGA-OR-A5K2-01A	TLCD2
LRP5	Gao et al	BLCA	TCGA-XF-AAML-01A	RBFOX1
LRP5	TumorFusions	BRCA	TCGA-BH-A18L-01A	NOX4
LRP5	TumorFusions	BRCA	TCGA-PE-A5DC-01A	ALKBH5
LRP5	TumorFusions	BRCA	TCGA-LD-A7W6-01A	C11orf30
LRP5	TumorFusions	BRCA	TCGA-BH-A0C3-01A	PPFIA1
LRP5	Gao et al	BRCA	TCGA-A1-A0SQ-01A	MOGAT2

LRP5	Gao et al	BRCA	TCGA-A2-A0CT-01A	C11orf80
LRP5	Gao et al	BRCA	TCGA-A2-A0CT-01A	RANGRF
LRP5	Gao et al	BRCA	TCGA-A2-A0D0-01A	ALG1L8P
LRP5	Gao et al	BRCA	TCGA-A8-A09I-01A	DLG2
LRP5	Gao et al	BRCA	TCGA-BH-A0C3-01A	PPFIA1
LRP5	Gao et al	BRCA	TCGA-BH-A0W3-01A	CASQ2
LRP5	Gao et al	BRCA	TCGA-BH-A18L-01A	NOX4
LRP5	Gao et al	BRCA	TCGA-BH-A18R-01A	DRAP1
LRP5	Gao et al	BRCA	TCGA-E2-A150-01A	ZNF507
LRP5	Gao et al	BRCA	TCGA-LD-A7W6-01A	EMSY
LRP5	Gao et al	BRCA	TCGA-LL-A6FQ-01A	MADD
LRP5	Gao et al	BRCA	TCGA-PE-A5DC-01A	ALKBH5
LRP5	TumorFusions	GBM	TCGA-06-2561-01A	ATG16L2
LRP5	Gao et al	GBM	TCGA-06-2561-01A	ATG16L2
LRP5	TumorFusions	HNSC	TCGA-IQ-7631-01A	ZNF317
LRP5	TumorFusions	HNSC	TCGA-CV-7418-01A	CLPB
LRP5	Gao et al	HNSC	TCGA-CV-6939-01A	HSCB
LRP5	Gao et al	HNSC	TCGA-CV-7418-01A	CLPB
LRP5	Gao et al	HNSC	TCGA-IQ-7631-01A	ZNF317
LRP5	TumorFusions	LGG	TCGA-HT-7686-01A	VPS37C
LRP5	Gao et al	LIHC	TCGA-DD-AADY-01A	MRPL48
LRP5	TumorFusions	LUAD	TCGA-97-A4M3-01A	CHD6
LRP5	TumorFusions	LUAD	TCGA-91-6831-01A	MRPL21
LRP5	TumorFusions	LUAD	TCGA-73-4658-01A	VPS26B
LRP5	Gao et al	LUAD	TCGA-91-6831-01A	MRPL21
LRP5	Gao et al	LUAD	TCGA-97-8179-01A	OR4G4P
LRP5	Gao et al	LUAD	TCGA-97-A4M3-01A	CHD6
LRP5	TumorFusions	LUSC	TCGA-85-8352-01A	SRPK2
LRP5	Gao et al	LUSC	TCGA-85-8352-01A	SRPK2
LRP5	TumorFusions	SKCM	TCGA-EB-A57M-01A	UVRAG
LRP5	Gao et al	SKCM	TCGA-EB-A57M-01A	UVRAG
LRP5	Gao et al	SKCM	TCGA-ER-A19T-01A	SMAD3
LRP5	Gao et al	STAD	TCGA-HU-A4GH-01A	CHRM1
LRP5	Gao et al	STAD	TCGA-HU-A4GH-01A	SLC22A6
MDH2	Gao et al	BRCA	TCGA-BH-A18H-01A	POR
MDH2	Gao et al	BRCA	TCGA-E2-A1L8-01A	PAWR
MDH2	Gao et al	LUSC	TCGA-22-4591-01A	STYXL1
MDH2	Gao et al	OV	TCGA-59-2348-01A	POR
MDH2	Gao et al	SARC	TCGA-DX-A240-01A	FGL2
DPF2	TumorFusions	GBM	TCGA-06-5415-01A	METTL12
DPF2	TumorFusions	GBM	TCGA-06-0141-01A	POLA2
DPF2	TumorFusions	LIHC	TCGA-DD-A11C-01A	FRMD8
DPF2	Gao et al	LIHC	TCGA-DD-A11C-01A	FRMD8
DPF2	TumorFusions	LUAD	TCGA-93-A4JO-01A	FRMD8

DPF2	Gao et al	LUAD	TCGA-93-A4JO-01A	FRMD8
DPF2	TumorFusions	LUSC	TCGA-85-8276-01A	AHNAK
DPF2	TumorFusions	LUSC	TCGA-52-7810-01A	CDC42EP2
DPF2	Gao et al	LUSC	TCGA-52-7810-01A	CDC42EP2
DPF2	Gao et al	LUSC	TCGA-85-8276-01A	AHNAK
DPF2	Gao et al	LUSC	TCGA-NC-A5HL-01A	CFL1
DPF2	Gao et al	OV	TCGA-29-1697-01A	MALAT1
DPF2	TumorFusions	SKCM	TCGA-W3-A825-06A	PPP1R10
DPF2	Gao et al	SKCM	TCGA-W3-A825-06A	PPP1R10
RPS27A	Gao et al	BRCA	TCGA-BH-A0RX-01A	MS4A4A
FIG4	TumorFusions	BRCA	TCGA-AO-A0J2-01A	ZNF292
FIG4	TumorFusions	BRCA	TCGA-BH-A203-01A	+
FIG4	Gao et al	BRCA	TCGA-AO-A0J2-01A	ZNF292
FIG4	Gao et al	BRCA	TCGA-AO-A124-01A	WASF1
FIG4	Gao et al	BRCA	TCGA-BH-A203-01A	WASF1
FIG4	TumorFusions	LAML	TCGA-AB-2901-03A	SEC63
FIG4	TumorFusions	LIHC	TCGA-2Y-A9HA-01A	HS3ST5
FIG4	Gao et al	PRAD	TCGA-J9-A8CL-01A	CCDC162P
FIG4	TumorFusions	SKCM	TCGA-FS-A1Z0-06A	PPP1R14C
FIG4	Gao et al	SKCM	TCGA-FS-A1Z0-06A	PPP1R14C
FIG4	TumorFusions	UCS	TCGA-NA-A5I1-01A	AMD1
FIG4	Gao et al	UCS	TCGA-NA-A5I1-01A	AMD1
CDH23	TumorFusions	BRCA	TCGA-BH-A18U-01A	BTRC
CDH23	TumorFusions	BRCA	TCGA-AN-A0FJ-01A	NSD1
CDH23	TumorFusions	BRCA	TCGA-AN-A0XN-01A	LTBP1
CDH23	Gao et al	BRCA	TCGA-A7-A26G-01A	MCU
CDH23	Gao et al	BRCA	TCGA-AN-A0FJ-01A	NSD1
CDH23	Gao et al	BRCA	TCGA-AN-A0FJ-01A	NSD1
CDH23	Gao et al	BRCA	TCGA-AN-A0XN-01A	LTBP1
CDH23	Gao et al	BRCA	TCGA-AN-A0XN-01A	LTBP1
CDH23	TumorFusions	LUAD	TCGA-78-8662-01A	CHST3
CDH23	TumorFusions	PRAD	TCGA-G9-6361-01A	C11orf30
CDH23	Gao et al	PRAD	TCGA-G9-6361-01A	EMSY
CDH23	TumorFusions	STAD	TCGA-CG-4449-01A	ASCC1
BAZ1B	TumorFusions	LIHC	TCGA-EP-A2KA-01A	MLXIPL
BAZ1B	TumorFusions	LIHC	TCGA-XR-A8TG-01A	BCL7B
BAZ1B	Gao et al	LIHC	TCGA-EP-A2KA-01A	MLXIPL
BAZ1B	Gao et al	LIHC	TCGA-XR-A8TG-01A	BCL7B
BAZ1B	Gao et al	OV	TCGA-13-0893-01B	BCL7B
BAZ1B	TumorFusions	PRAD	TCGA-HC-8265-01A	POMZP3
BAZ1B	TumorFusions	PRAD	TCGA-HC-8265-01A	ZP3
BAZ1B	Gao et al	PRAD	TCGA-HC-8265-01A	POMZP3
BAZ1B	Gao et al	PRAD	TCGA-HC-8265-01A	ZP3
BAZ1B	Gao et al	PRAD	TCGA-M7-A725-01A	TYW1B

BAZ1B	TumorFusions	STAD	TCGA-CG-4443-01A	MLXIPL
BAZ1B	TumorFusions	UCEC	TCGA-EY-A54A-01A	NSUN5
BAZ1B	TumorFusions	UCEC	TCGA-EY-A2OQ-01A	EDEM3
BAZ1B	Gao et al	UCEC	TCGA-EY-A54A-01A	NSUN5
BAZ1B	Gao et al	UCEC	TCGA-KP-A3W1-01A	BCL7B
XRCC4	TumorFusions	BRCA	TCGA-A8-A07C-01A	PIGU
XRCC4	Gao et al	BRCA	TCGA-A8-A07C-01A	PIGU
XRCC4	TumorFusions	LUSC	TCGA-43-3920-01A	COL4A5
XRCC4	TumorFusions	SARC	TCGA-K1-A6RV-01A	RASA1
XRCC4	Gao et al	SARC	TCGA-K1-A6RV-01A	RASA1
GBA	TumorFusions	BRCA	TCGA-A2-A04U-01A	POGZ
GBA	Gao et al	BRCA	TCGA-A2-A04U-01A	POGZ
GBA	Gao et al	HNSC	TCGA-D6-8568-01A	OTUB1
GBA	TumorFusions	LIHC	TCGA-KR-A7K8-01A	S100A13
GBA	Gao et al	LIHC	TCGA-KR-A7K8-01A	S100A13
GBA	TumorFusions	UCEC	TCGA-BK-A139-02A	MUC1
GPC4	Gao et al	LIHC	TCGA-2Y-A9GU-01A	GPC3
GPC4	Gao et al	LUSC	TCGA-39-5029-01A	IGHJ4
GPC4	TumorFusions	TGCT	TCGA-2G-AAKD-01A	GPC3
ARX	TumorFusions	LGG	TCGA-DU-6404-02A	SCO1
CLIP2	TumorFusions	BRCA	TCGA-AO-A126-01A	MLL3
CLIP2	TumorFusions	SKCM	TCGA-EE-A29L-06A	RORB
CLIP2	Gao et al	SKCM	TCGA-YG-AA3O-06A	GTF2I
CLIP2	Gao et al	STAD	TCGA-VQ-A8PK-01A	GTF2I
CLIP2	TumorFusions	UCEC	TCGA-D1-A3JP-01A	MTMR3
CLIP2	Gao et al	UCEC	TCGA-D1-A3DG-01A	NCF1B
CLIP2	Gao et al	UCEC	TCGA-D1-A3JP-01A	MTMR3
CLIP2	TumorFusions	UCS	TCGA-N9-A4Q1-01A	GTF2IRD1
CLIP2	Gao et al	UCS	TCGA-ND-A4WA-01A	PMS2P1
PAK3	TumorFusions	CESC	TCGA-IR-A3LB-01A	SH2D1A
PAK3	Gao et al	GBM	TCGA-76-4926-01B	STAG2
TNFRSF1A	TumorFusions	CESC	TCGA-VS-A94W-01A	SCNN1A
TNFRSF1A	Gao et al	OV	TCGA-23-1111-01A	PCBP2
TNFRSF1A	TumorFusions	SKCM	TCGA-D3-A2JN-06A	CHD4
IHH	Gao et al	STAD	TCGA-BR-8289-01A	NHEJ1
BMPR1B	TumorFusions	BRCA	TCGA-A2-A0ET-01A	MCC
BMPR1B	Gao et al	BRCA	TCGA-A2-A0ET-01A	MCC
BMPR1B	TumorFusions	KIRC	TCGA-B0-4698-01A	DDAH1
BMPR1B	TumorFusions	LUAD	TCGA-44-7670-01A	NSUN4
BMPR1B	TumorFusions	PRAD	TCGA-HC-A4ZV-01A	PDLIM5
BMPR1B	TumorFusions	PRAD	TCGA-EJ-7330-01A	PDLIM5
BMPR1B	TumorFusions	PRAD	TCGA-SU-A7E7-01A	ETV1
BMPR1B	Gao et al	PRAD	TCGA-SU-A7E7-01A	ETV1
TBL2	TumorFusions	LIHC	TCGA-DD-A115-01A	BCL7B

FBXW11	Gao et al	BRCA	TCGA-AC-A8OQ-01A	UBTD2
FBXW11	TumorFusions	CESC	TCGA-VS-A9UT-01A	KCNIP1
FBXW11	Gao et al	CESC	TCGA-VS-A9UT-01A	KCNIP1
FBXW11	TumorFusions	LIHC	TCGA-NI-A4U2-01A	NUP210L
FBXW11	Gao et al	LIHC	TCGA-NI-A4U2-01A	NUP210L
FBXW11	Gao et al	SARC	TCGA-FX-A2QS-01A	LMAN2
FBXW11	TumorFusions	SKCM	TCGA-D3-A8GN-06A	RBM11
FBXW11	Gao et al	SKCM	TCGA-D3-A51N-06A	TTC33
COL1A2	TumorFusions	BRCA	TCGA-AC-A2QH-01B	WNT2
COL1A2	Gao et al	BRCA	TCGA-A8-A09R-01A	PEG10
COL1A2	Gao et al	BRCA	TCGA-E9-A1NF-01A	POSTN
COL1A2	Gao et al	CESC	TCGA-VS-A9UH-01A	HSPA8
COL1A2	Gao et al	LUAD	TCGA-J2-8192-01A	NAPSA
COL1A2	Gao et al	LUSC	TCGA-85-6175-01A	P4HB
COL1A2	Gao et al	PAAD	TCGA-IB-A5SS-01A	SERPINE1
COL1A2	Gao et al	SARC	TCGA-DX-A3UA-01A	CD63
COL1A2	Gao et al	SARC	TCGA-QQ-A8VD-01A	SHC1
COL1A2	Gao et al	SARC	TCGA-SI-A71O-01A	CPOX
COL1A2	Gao et al	SARC	TCGA-SI-A71O-01A	POSTN
COL1A2	Gao et al	SARC	TCGA-SI-AA8C-01A	ITM2C
COL1A2	Gao et al	UCS	TCGA-NA-A4QV-01A	FSTL1
DVL3	TumorFusions	ESCA	TCGA-R6-A8W5-01B	RFX6
DVL3	Gao et al	ESCA	TCGA-R6-A8W5-01B	RFX6
DVL3	Gao et al	LUSC	TCGA-56-8628-01A	VPS8
DVL3	Gao et al	OV	TCGA-57-1994-01A	ECE2
GAB1	Gao et al	BRCA	TCGA-BH-A18V-01A	DDR2
GAB1	Gao et al	ESCA	TCGA-Q9-A6FU-01A	PPP2R2C
GAB1	Gao et al	KIRC	TCGA-CZ-5469-01A	FAM13A
GAB1	TumorFusions	PRAD	TCGA-VN-A943-01A	GALNT7
GAB1	Gao et al	PRAD	TCGA-VN-A943-01A	GALNT7
GDF5	TumorFusions	LUSC	TCGA-46-6025-01A	UQCC
GDF5	Gao et al	LUSC	TCGA-46-6025-01A	UQCC1
POLR3A	Gao et al	BRCA	TCGA-AN-A0AJ-01A	TP53BP2
POLR3A	TumorFusions	LUSC	TCGA-85-7697-01A	DLG5
POLR3A	Gao et al	PRAD	TCGA-ZG-A9N3-01A	TTC6
POLR3A	Gao et al	PRAD	TCGA-ZG-A9N3-01A	TTC6
POLR3A	Gao et al	SARC	TCGA-DX-A23Z-01A	SASH1
POLR3A	TumorFusions	UCS	TCGA-N5-A59F-01A	CUEDC2
POLR3A	Gao et al	UCS	TCGA-N5-A59F-01A	CUEDC2
KIF1A	TumorFusions	LUAD	TCGA-NJ-A4YF-01A	C2orf54
KIF1A	Gao et al	LUAD	TCGA-NJ-A4YF-01A	C2orf54
KIF1A	TumorFusions	LUSC	TCGA-46-6026-01A	TRIP12
KIF1A	Gao et al	LUSC	TCGA-46-6026-01A	TRIP12
KIF1A	TumorFusions	UCEC	TCGA-A5-A7WK-01A	C2orf54

KIF1A	Gao et al	UCEC	TCGA-A5-A7WK-01A	C2orf54
STXBP1	TumorFusions	READ	TCGA-AG-3731-01A	SUV420H1
STXBP1	Gao et al	READ	TCGA-AG-3731-01A	KMT5B
STXBP1	TumorFusions	UCEC	TCGA-A5-A7WK-01A	CACNA2D2
STXBP1	TumorFusions	UCEC	TCGA-A5-A7WK-01A	LUC7L
STXBP1	Gao et al	UCEC	TCGA-A5-A7WK-01A	LUC7L
STXBP1	Gao et al	UCEC	TCGA-A5-A7WK-01A	CACNA2D2
STXBP1	Gao et al	UCEC	TCGA-AJ-A3BF-01A	CERCAM
STXBP1	Gao et al	UCEC	TCGA-B5-A5OE-01A	LRSAM1
STXBP1	TumorFusions	UCS	TCGA-N8-A56S-01A	SOX13
STXBP1	Gao et al	UCS	TCGA-NA-A4R1-01A	RALGPS1

Table S9 Functional enrichment analysis results of 20/20plus-predicted cancer genes using DAVID.

Term category	Term	Count	FDR
GO_BP	GO:0006511~ubiquitin-dependent protein catabolic process	21	7.60E-09
	GO:0045893~positive regulation of transcription, DNA-templated	28	1.07E-06
	GO:0048511~rhythmic process	10	4.11E-05
	GO:0016567~protein ubiquitination	20	1.22E-04
	GO:0045892~negative regulation of transcription, DNA-templated	20	1.21E-03
	GO:0006974~cellular response to DNA damage stimulus	14	1.56E-03
	GO:0007049~cell cycle	15	2.60E-03
	GO:0006325~chromatin organization	14	2.68E-03
	GO:0045944~positive regulation of transcription from RNA polymerase II promoter	28	7.23E-03
	GO:0006468~protein phosphorylation	15	1.27E-02
GO_CC	GO:0005829~cytosol	113	1.87E-18
	GO:0005654~nucleoplasm	84	2.77E-12
	GO:0005634~nucleus	106	3.47E-12
	GO:0005737~cytoplasm	88	4.40E-06
	GO:0032991~macromolecular complex	22	1.20E-04
	GO:0048471~perinuclear region of cytoplasm	22	3.40E-04
	GO:0042995~cell projection	11	8.44E-04
	GO:0000785~chromatin	26	1.26E-03
	GO:0030054~cell junction	11	2.69E-03
	GO:0005925~focal adhesion	14	6.35E-03
GO_MF	GO:0005515~protein binding	182	3.59E-22
	GO:0003723~RNA binding	38	2.58E-05
	GO:0004842~ubiquitin-protein transferase activity	15	3.01E-05
	GO:0061630~ubiquitin protein ligase activity	16	2.87E-04
	GO:0005524~ATP binding	35	5.92E-04
	GO:0005178~integrin binding	10	2.23E-03

	GO:0003713~transcription coactivator activity	12	4.50E-03
	GO:0008022~protein C-terminus binding	10	6.64E-03
	GO:0004712~protein serine/threonine/tyrosine kinase activity	15	6.64E-03
	GO:0003779~actin binding	13	6.81E-03
KEGG PATHWAY	hsa04120:Ubiquitin mediated proteolysis	11	1.25E-02
	hsa04530:Tight junction	11	2.80E-02
	hsa04151:PI3K-Akt signaling pathway	14	2.28E-01
	hsa04910:Insulin signaling pathway	8	2.72E-01
	hsa04810:Regulation of actin cytoskeleton	10	3.63E-01
	hsa05165:Human papillomavirus infection	12	3.93E-01
	hsa05205:Proteoglycans in cancer	9	3.93E-01
	hsa05200:Pathways in cancer	16	3.93E-01
	hsa00310:Lysine degradation	5	3.93E-01
	hsa04928:Parathyroid hormone synthesis, secretion and action	6	5.20E-01
REACTOME PATHWAY	R-HSA-162582~Signal Transduction	68	1.97E-03
	R-HSA-983168~Antigen processing: Ubiquitination & Proteasome degradation	16	4.14E-02
	R-HSA-983169~Class I MHC mediated antigen processing & presentation	16	1.60E-01
	R-HSA-74160~Gene expression (Transcription)	40	1.60E-01
	R-HSA-3247509~Chromatin modifying enzymes	13	1.60E-01
	R-HSA-4839726~Chromatin organization	13	1.60E-01
	R-HSA-1280218~Adaptive Immune System	24	2.20E-01
	R-HSA-3108232~SUMO E3 ligases SUMOylate target proteins	10	2.39E-01
	R-HSA-212165~Epigenetic regulation of gene expression	10	2.39E-01
	R-HSA-1500931~Cell-Cell communication	9	2.39E-01

Table S10 Functional enrichment analysis results of DORGE-predicted cancer genes using DAVID.

Term category	Term	Count	FDR
GO_BP	GO:0006468~protein phosphorylation	24	5.96E-08
	GO:0045944~positive regulation of transcription from RNA polymerase II promoter	33	1.04E-04
	GO:0018105~peptidyl-serine phosphorylation	13	1.04E-04
	GO:0035556~intracellular signal transduction	19	1.72E-04
	GO:0016310~phosphorylation	15	2.42E-04
	GO:1900181~negative regulation of protein localization to nucleus	6	7.43E-04
	GO:0033674~positive regulation of kinase activity	8	1.27E-03
	GO:0048013~ephrin receptor signaling pathway	7	1.56E-03
	GO:0046777~protein autophosphorylation	11	1.72E-03
	GO:0006325~chromatin organization	14	2.06E-03
GO_CC	GO:0005829~cytosol	91	1.44E-07
	GO:0005654~nucleoplasm	73	2.07E-07

	GO:0005634~nucleus	94	2.72E-07
	GO:0005925~focal adhesion	19	6.75E-06
	GO:0030424~axon	17	1.81E-05
	GO:0098978~glutamatergic synapse	18	1.81E-05
	GO:0043235~receptor complex	12	3.07E-04
	GO:0043034~costamere	5	6.62E-04
	GO:0030425~dendrite	16	6.62E-04
	GO:0005886~plasma membrane	76	6.62E-04
GO_MF	GO:0004712~protein serine/threonine/tyrosine kinase activity	28	1.17E-11
	GO:0005515~protein binding	163	1.06E-08
	GO:0004674~protein serine/threonine kinase activity	22	5.22E-08
	GO:0005524~ATP binding	43	7.09E-08
	GO:0004672~protein kinase activity	20	5.46E-07
	GO:0003779~actin binding	18	2.98E-06
	GO:0003723~RNA binding	34	3.44E-04
	GO:0004714~transmembrane receptor protein tyrosine kinase activity	7	6.82E-04
	GO:0008017~microtubule binding	13	6.82E-04
	GO:0016301~kinase activity	12	7.19E-04
KEGG PATHWAY	hsa04360:Axon guidance	15	1.95E-05
	hsa04010:MAPK signaling pathway	14	1.48E-02
	hsa04380:Osteoclast differentiation	9	1.51E-02
	hsa05220:Chronic myeloid leukemia	7	1.51E-02
	hsa05010:Alzheimer disease	15	1.51E-02
	hsa01521:EGFR tyrosine kinase inhibitor resistance	7	1.51E-02
	hsa05017:Spinocerebellar ataxia	9	1.51E-02
	hsa04722:Neurotrophin signaling pathway	8	1.74E-02
	hsa05167:Kaposi sarcoma-associated herpesvirus infection	10	1.74E-02
	hsa04919:Thyroid hormone signaling pathway	8	1.74E-02
REACTOME PATHWAY	R-HSA-9675108~Nervous system development	28	1.40E-05
	R-HSA-162582~Signal Transduction	66	3.23E-05
	R-HSA-422475~Axon guidance	26	3.23E-05
	R-HSA-1266738~Developmental Biology	40	1.52E-04
	R-HSA-5663202~Diseases of signal transduction by growth factor receptors and second messengers	21	7.01E-04
	R-HSA-2682334~EPH-Ephrin signaling	9	4.48E-03
	R-HSA-9006934~Signaling by Receptor Tyrosine Kinases	21	4.48E-03
	R-HSA-3928662~EPHB-mediated forward signaling	6	2.72E-02
	R-HSA-3928665~EPH-ephrin mediated repulsion of cells	6	6.05E-02
	R-HSA-73857~RNA Polymerase II Transcription	34	6.83E-02

Table S11 Functional enrichment analysis results of EMOGI-predicted cancer genes using DAVID.

Term category	Term	Count	FDR

GO_BP	GO:0034613~cellular protein localization	10	3.60E-05
	GO:0006338~chromatin remodeling	18	3.60E-05
	GO:0007155~cell adhesion	22	9.63E-05
	GO:0000226~microtubule cytoskeleton organization	12	9.63E-05
	GO:0006974~cellular response to DNA damage stimulus	16	1.02E-04
	GO:0043066~negative regulation of apoptotic process	21	1.18E-04
	GO:0008284~positive regulation of cell proliferation	21	1.18E-04
	GO:0018107~peptidyl-threonine phosphorylation	9	1.30E-04
	GO:0006468~protein phosphorylation	18	4.04E-04
	GO:0009410~response to xenobiotic stimulus	14	6.24E-04
GO_CC	GO:0005829~cytosol	121	5.21E-23
	GO:0005737~cytoplasm	115	1.67E-18
	GO:0005925~focal adhesion	29	5.32E-14
	GO:0070062~extracellular exosome	59	8.10E-12
	GO:0030018~Z disc	17	1.02E-11
	GO:0005634~nucleus	103	1.15E-10
	GO:0005886~plasma membrane	94	8.85E-10
	GO:0042383~sarcolemma	14	1.13E-09
	GO:0032991~macromolecular complex	29	2.12E-09
	GO:0005938~cell cortex	16	4.61E-09
GO_MF	GO:0045296~cadherin binding	23	5.32E-10
	GO:0019899~enzyme binding	24	1.51E-09
	GO:0005515~protein binding	167	2.43E-09
	GO:0051015~actin filament binding	19	2.43E-09
	GO:0031625~ubiquitin protein ligase binding	21	5.04E-09
	GO:0019901~protein kinase binding	25	3.27E-08
	GO:0042802~identical protein binding	47	3.27E-08
	GO:0019904~protein domain specific binding	18	3.70E-08
	GO:0005200~structural constituent of cytoskeleton	13	4.69E-08
	GO:0005509~calcium ion binding	29	9.29E-08
KEGG PATHWAY	hsa05203:Viral carcinogenesis	19	1.10E-06
	hsa05161:Hepatitis B	15	3.70E-05
	hsa04510:Focal adhesion	16	6.02E-05
	hsa05205:Proteoglycans in cancer	16	6.02E-05
	hsa04110:Cell cycle	14	6.28E-05
	hsa04062:Chemokine signaling pathway	15	9.49E-05
	hsa04010:MAPK signaling pathway	18	2.32E-04
	hsa04540:Gap junction	10	2.87E-04
	hsa04151:PI3K-Akt signaling pathway	19	4.93E-04
	hsa05165:Human papillomavirus infection	17	1.95E-03
REACTOME PATHWAY	R-HSA-162582~Signal Transduction	82	2.02E-10
	R-HSA-1500931~Cell-Cell communication	20	3.87E-10
	R-HSA-9006934~Signaling by Receptor Tyrosine Kinases	30	2.40E-07
	R-HSA-9675108~Nervous system development	29	4.93E-06

	R-HSA-9679506~SARS-CoV Infections	24	7.06E-06
	R-HSA-76002~Platelet activation, signaling and aggregation	19	9.01E-06
	R-HSA-1643685~Disease	55	1.33E-05
	R-HSA-446728~Cell junction organization	13	1.33E-05
	R-HSA-5663202~Diseases of signal transduction by growth factor receptors and second messengers	24	2.36E-05
	R-HSA-9680350~Signaling by CSF1 (M-CSF) in myeloid cells	8	2.36E-05

Table S12 Functional enrichment analysis results of MTGCN-predicted cancer genes using DAVID.

Term category	Term	Count	FDR
GO_BP	GO:0007169~transmembrane receptor protein tyrosine kinase signaling pathway	18	1.02E-10
	GO:0006468~protein phosphorylation	28	1.02E-10
	GO:0007165~signal transduction	46	2.23E-10
	GO:0007155~cell adhesion	30	4.30E-10
	GO:0016477~cell migration	20	3.26E-08
	GO:0098609~cell-cell adhesion	17	6.24E-08
	GO:0043410~positive regulation of MAPK cascade	16	8.22E-08
	GO:0030335~positive regulation of cell migration	19	8.22E-08
	GO:0051897~positive regulation of protein kinase B signaling	16	1.87E-07
	GO:0038096~Fc-gamma receptor signaling pathway involved in phagocytosis	8	2.42E-07
GO_CC	GO:0005925~focal adhesion	39	4.94E-23
	GO:0005886~plasma membrane	121	5.03E-22
	GO:0005911~cell-cell junction	27	1.94E-20
	GO:0070062~extracellular exosome	74	2.66E-20
	GO:0005737~cytoplasm	120	1.43E-19
	GO:0005829~cytosol	115	1.06E-17
	GO:0042383~sarcolemma	20	3.79E-17
	GO:0045121~membrane raft	26	7.99E-17
	GO:0005856~cytoskeleton	35	2.14E-16
	GO:0005938~cell cortex	21	2.75E-14
GO_MF	GO:0005515~protein binding	182	6.63E-14
	GO:0042802~identical protein binding	59	1.05E-13
	GO:0019899~enzyme binding	29	1.08E-13
	GO:0019901~protein kinase binding	32	4.58E-13
	GO:0005178~integrin binding	20	5.10E-13
	GO:0045296~cadherin binding	25	3.37E-12
	GO:0004712~protein serine/threonine/tyrosine kinase activity	28	1.09E-11
	GO:0051015~actin filament binding	20	2.98E-10
	GO:0005200~structural constituent of cytoskeleton	15	4.12E-10
	GO:0001784~phosphotyrosine binding	11	2.72E-09
KEGG	hsa04510:Focal adhesion	28	6.62E-14

PATHWAY	hsa04062:Chemokine signaling pathway	26	6.72E-13
	hsa05205:Proteoglycans in cancer	26	2.10E-12
	hsa05135:Yersinia infection	21	2.06E-11
	hsa05131:Shigellosis	26	8.95E-11
	hsa04010:MAPK signaling pathway	27	9.91E-10
	hsa04611:Platelet activation	18	1.90E-09
	hsa05163:Human cytomegalovirus infection	22	1.53E-08
	hsa04670:Leukocyte transendothelial migration	16	3.89E-08
	hsa04380:Osteoclast differentiation	17	4.26E-08
REACTOME PATHWAY	R-HSA-162582~Signal Transduction	105	4.99E-22
	R-HSA-9006934~Signaling by Receptor Tyrosine Kinases	48	1.57E-20
	R-HSA-76002~Platelet activation, signaling and aggregation	36	1.57E-20
	R-HSA-109582~Hemostasis	46	2.84E-16
	R-HSA-1280215~Cytokine Signaling in Immune system	47	4.10E-13
	R-HSA-194138~Signaling by VEGF	20	6.02E-13
	R-HSA-449147~Signaling by Interleukins	36	1.25E-12
	R-HSA-4420097~VEGFA-VEGFR2 Pathway	19	1.37E-12
	R-HSA-9680350~Signaling by CSF1 (M-CSF) in myeloid cells	13	1.71E-12
	R-HSA-5673001~RAF/MAP kinase cascade	28	2.55E-12

Table S13 Functional enrichment analysis results of NetCore-predicted cancer genes using DAVID.

Term category	Term	Count	FDR
GO_BP	GO:0045944~positive regulation of transcription from RNA polymerase II promoter	28	5.40E-18
	GO:0000122~negative regulation of transcription from RNA polymerase II promoter	22.5	1.73E-13
	GO:0045893~positive regulation of transcription, DNA-templated	19	3.73E-13
	GO:0043161~proteasome-mediated ubiquitin-dependent protein catabolic process	11	8.04E-12
	GO:0045892~negative regulation of transcription, DNA-templated	16	2.85E-11
	GO:0006468~protein phosphorylation	14	3.49E-11
	GO:0006511~ubiquitin-dependent protein catabolic process	11	6.86E-10
	GO:0016575~histone deacetylation	6	1.34E-09
	GO:0018105~peptidyl-serine phosphorylation	9	1.62E-09
	GO:0016567~protein ubiquitination	13.5	2.08E-09
GO_CC	GO:0005654~nucleoplasm	65.5	2.74E-42
	GO:0005634~nucleus	77.5	3.18E-42
	GO:0005829~cytosol	72.5	3.70E-38
	GO:0005737~cytoplasm	67	2.36E-28
	GO:0032991~macromolecular complex	19.5	1.95E-16
	GO:0070062~extracellular exosome	33.5	1.06E-15

	GO:1990904~ribonucleoprotein complex	10	2.11E-12
	GO:0005925~focal adhesion	13.5	5.78E-12
	GO:0016605~PML body	7.5	1.45E-10
	GO:0000785~chromatin	18.5	5.61E-09
GO_MF	GO:0019899~enzyme binding	24	6.37E-34
	GO:0031625~ubiquitin protein ligase binding	21.5	6.16E-32
	GO:0005515~protein binding	99.5	1.04E-31
	GO:0042802~identical protein binding	33	1.17E-18
	GO:0019904~protein domain specific binding	13.5	1.35E-16
	GO:0019901~protein kinase binding	17	5.68E-15
	GO:0003723~RNA binding	27.5	9.54E-15
	GO:0003713~transcription coactivator activity	10	3.49E-09
	GO:1990841~promoter-specific chromatin binding	6	5.22E-09
	GO:0061629~RNA polymerase II sequence-specific DNA binding transcription factor binding	8.5	6.42E-09
KEGG PATHWAY	hsa05203:Viral carcinogenesis	15.5	3.65E-16
	hsa05131:Shigellosis	14.5	2.84E-12
	hsa05160:Hepatitis C	11.5	1.89E-11
	hsa05417:Lipid and atherosclerosis	13	2.10E-11
	hsa05135:Yersinia infection	10.5	5.75E-11
	hsa05170:Human immunodeficiency virus 1 infection	12.5	7.61E-11
	hsa05161:Hepatitis B	10.5	9.69E-10
	hsa04120:Ubiquitin mediated proteolysis	9.5	5.35E-09
	hsa04114:Oocyte meiosis	9	1.05E-08
	hsa05130:Pathogenic Escherichia coli infection	10.5	2.55E-08
REACTOME PATHWAY	R-HSA-1643685~Disease	52	4.07E-30
	R-HSA-5663205~Infectious disease	38.5	8.56E-28
	R-HSA-162582~Signal Transduction	58.5	3.44E-26
	R-HSA-9824446~Viral Infection Pathways	34	3.44E-26
	R-HSA-1280215~Cytokine Signaling in Immune system	30	5.83E-21
	R-HSA-9679506~SARS-CoV Infections	21.5	4.44E-19
	R-HSA-449147~Signaling by Interleukins	22.5	1.17E-18
	R-HSA-597592~Post-translational protein modification	38	8.39E-18
	R-HSA-168164~Toll Like Receptor 3 (TLR3) Cascade	12	1.78E-17
	R-HSA-168256~Immune System	45	3.08E-17

Table S14 Comparative experiment between GCN, HGNN and DISHyper.

Method	AUROC	AUPRC
GCN	0.907 ± 0.017	0.836 ± 0.029
HGNN	0.913 ± 0.016	0.861 ± 0.021
DISHyper	0.937 ± 0.014	0.894 ± 0.019
